# Cannabinoid as Beneficial Replacement Therapy for Psychotropics to Treat Neuropsychiatric Symptoms in Severe Alzheimer’s Dementia: A Clinical Case Report

**DOI:** 10.3389/fpsyt.2020.00413

**Published:** 2020-05-13

**Authors:** Michaela Defrancesco, Alex Hofer

**Affiliations:** Department of Psychiatry, Psychotherapy and Psychosomatics, Division of Psychiatry I, Medical University of Innsbruck, Innsbruck, Austria

**Keywords:** Alzheimer’s disease, dronabinol, neuropsychiatric symptoms, psychotropics, dementia

## Abstract

Alzheimer’s Dementia (AD) is a devastating neurodegenerative disease that affects approximately 17% of people aged 75–84. Neuropsychiatric symptoms (NPS) such as delusions, agitation, anxiety, and hallucinations are present in up to 95% of patients in all stages of dementia. To date, any approved and effective pharmacological interventions for the treatment of NPS are still not available. We describe a clinical case of a female patient diagnosed with AD with continuous cognitive decline and dementia-related behavioral symptoms. Between 2008 and 2019, the patient was examined half-yearly at the memory clinic of the Medical University of Innsbruck. At each visit, cognitive state and pharmacological treatment were evaluated. In addition, NPs were assessed by using the neuropsychiatric inventory (NPI). In 2018, the patient progressed to severe AD stage and presented with progressive NPs (anxiety, suspected delusions, agitation, aggressive behavior, and suspected pain due to long immobility). Consequently, off-label treatment with low-dose dronabinol was initiated, which facilitated a reduction of psychopharmacological treatment from six to three psychotropics. At the same time, the patient’s emotional state improved, while disruptive behavior, aggression, and sedation decreased significantly. This case report underpins the need for randomized, controlled trials to explore the effect of cannabinoid receptor agonists on behavioral and psychological symptoms in patients with severe AD.

## Introduction

In 2015, dementia of all aetiologies affected 47 million people worldwide and was predicted to increase to 75 million people until 2030 (1). Alzheimer’s Dementia (AD) is a devastating neurodegenerative disease and the most common form of dementia. AD contributes to 60%–70% of cases and affects approximately 17% of people aged 75–84 and 32% of people aged 85 or older (1, 2). Progressive impairment of memory and cognition is a key clinical feature of Alzheimer’s disease. It is characterized by extracellular beta-amyloid (Abeta 42) deposits in the brain (plaques), intraneuronal tau pathology, neuronal cell death, vascular dysfunction, and inflammatory processes (3, 4). Neuropsychiatric symptoms (NPS) such as delusions, agitation, anxiety, and hallucinations are common across all stages of dementia (5). NPS are associates with a more rapid disease progression (6) and poor caregiver outcome (7). Especially in severe stages of dementia the differentiation and assessment of underlying causes of NPS is challenging. Symptoms like anxiety, aggression, or agitation due to somatic reasons such as pain are frequently misinterpreted as dementia-related and are consequently consistently treated with psychotropics, although nonpharmacological medical care is recommended as first-line treatment. It has been suggested that NPS rather than cognitive or functional impairment lead to the greatest caregiver burden and predict an early institutionalisation of patients with dementia.

There is no FDA-approved pharmacotherapy for NPS, however, psychotropics such as antipsychotics or benzodiazepines are frequently used to control these symptoms. Results from randomized controlled trials on the efficacy and safety of pharmacological treatment of NPS are conflicting (8). Cholinesterase inhibitors and new-generation antipsychotics improve NPS, but high rates of adverse events have been reported. Similarly, the use of first-generation antipsychotics is associated with a significant risk for adverse events and increased mortality rates (9). As a consequence, regulatory agencies and most guidelines have advised against the use of antipsychotic drugs in patients with AD (10). Alternative pharmacological treatment options are therefore urgently needed. A small number of clinical trials and several case reports have shown that cannabinoids such as oral dronabinol (Δ^9^-tetrahydrocannabinol, THC) may be promising in this regard (11–14). Some *in vitro* studies in AD transgenic mice even reported on the neuroprotective and antiinflammatory properties of these drugs (15). To date, cannabinoid receptor agonists are commonly used to treat nausea, anorexia, pain, and anxiety in oncological patients and in those with severe spasticity. Serious side effects such as marked orthostatic hypotension or psychotic symptoms are rare, however, metabolization of THC by liver cytochromes P450 can cause considerable drug-interactions. Therefore, further investigations addressing the therapeutic potential of THC in patients with AD in clinical studies and pharmacological models are urgently needed. In this case report we describe a female patient in a severe stage of AD who presented with symptoms of anxiety, agitation, and disruptive behavior and whose condition improved significantly after implementing dronabinol as replacement therapy of prior psychotropic medication.

## Materials and Methods

This case report describes continuous cognitive decline, behavioral symptoms, and the course of pharmacological treatment in a female patient diagnosed with AD according to the original NINCDS-ADRDA criteria (1984) in 2008. At first admission at the Department of Psychiatry, Psychotherapy and Psychosomatics of the Medical University of Innsbruck in June 2008 the patient was 69 years of age and presented with mild depressive symptoms and subjective deficits in memory and spatial orientation. Her daughter also reported on progressive deficits in activities of daily living (housekeeping, budget management, cooking), and on episodes of paranoid perception starting within the year before admission. In addition, the patient had lost 10 kg of weight within the past three years despite a good appetite.

**Figure 1:**
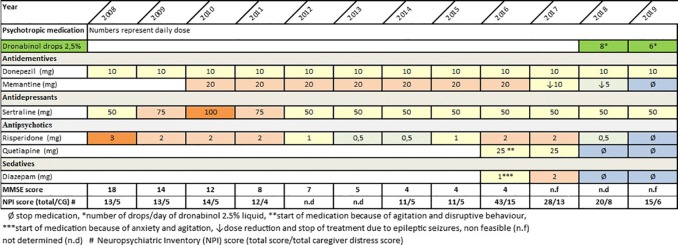
Course of psychotropic medication, cognitive decline and neuropsychiatric symptoms between time of Alzheimer’s Dementia (AD) diagnosis and August 2019.

Neuropsychological testing with the Mini Mental State Examination (MMSE) and “The Consortium on the Establishment of a Registry in Alzheimer’s Disease (CERAD)-Plus Neuropsychological Battery,” revealed an MMSE score of 18 and deficits in verbal and figural memory, psychomotor speed, executive functions, and verbal fluency. These results corresponded to a moderate stage of dementia. The Neuropsychiatric Inventory (NPI) (16) indicated mild symptoms of depression, anxiety, and delusion. Caregiver distress was low. To monitor frequency, severity and caregiver distress, the NPS total score and the caregiver distress subscore were calculated (see **Figure 1**). Magnetic resonance imaging (1.5 tesla) revealed gray matter atrophy in the mesial temporal lobe including the hippocampus, in the insula, and in the parietal cortex. Laboratory results (blood cell count, kidney and liver function, lipid profile, thyroid function, electrolytes, and levels of folic acid and vitamin B12) were unremarkable. The Apo E4 genotype was 4/3. Prediagnosed mild hypertension and type II diabetes mellitus were treated with metformin 1,000 mg/day and enalapril 5 mg/day. In addition, the patients received oral supplement of Vitamin D3 (15 drops/week) for the prophylaxis of osteoporosis. Family history was free of dementia and cerebro- and cardiovascular events. The patient had two children (one son and one daughter), 12 years of education, and she had been employed as a secretary until 1999. Based on the results of neuropsychological assessment, neuroimaging and clinical examination AD and mild to moderate depression (ICD-10) were diagnosed and treatment with donepezil 5 mg/day (10 mg/day from week 4 on), risperidone 3 mg/day, and sertraline 50 mg/day was started in August 2008. Due to rapid cognitive decline, persisting behavioral symptoms (agitation, disruptive behavior), and progressive deficits in activities of daily living, additional treatment with memantine (titrated to 20 mg/day within 4 weeks) was implemented in February 2010. Subsequently, behavioral symptoms improved and the dose of risperidone could be reduced to 0.5 mg/day. Treatment with donepezil 10 mg/day was continued. In June 2015, the patient progressed to severe dementia stage and behavioral symptoms (agitation, disruptive behavior, anxiety) worsened again. This led to substantial difficulties in nursing care and consequently, the treating general practitioner augmented psychopharmacological treatment with quetiapine 25 mg/day and diazepam 1mg/day in January 2016. Concurrently, the dose of risperidone was increased to 2 mg/day.

Within the following year, a number of adverse events due to psychotropic polypharmacy occurred. The patient lost mobility, suffered from sedation, spontaneous speech stopped, and emotional reactions suspended. In consequence of frequently occurring disruptive behavior and physical aggression during nursing care, treating the patient at home became more and more difficult. Moreover, a first epileptic seizure most likely caused by AD pathology occurred in March 2017. Following three further epileptic seizures in June 2017, treatment with levetiracetam up to 1,000 mg/day was started. Concurrently, the dose of memantine was reduced to 5 mg/day in order to reduce the risk of epileptic seizures Extensive polypharmacy including seven psychotropic agents, immobility and severe impairments on a cognitive, a psychopathological and an emotional level made an immediate reevaluation of drug therapy indispensable. The patient’s low quality of life and a presumed high degree of psychological and physical burden (anxiety, suspected delusions, suspected pain due to long immobility) led to a consequent tapering of psychotropic medication and to the implementation of dronabinol (magistral prescription, Δ^9^-tetrahydrocannabinol, 0.833 mg dronabinol/drop) as off-label therapy in February 2018. Benefits and risks of dronabinol as off-label treatment were fully explained to both the patient and her family members, and verbal consent was obtained from the family members. Caregivers were informed on potential side effects such as hypotension, psychotic symptoms, and sedation and possible drug-interactions with other drugs metabolized by the liver e.g., anticoagulants and a number of antibiotics. Furthermore, the expected benefit of low-dose dronabinol, i.e., a reduction of disruptive behavior, agitation, and anxiety symptoms without sedation and without an increased risk of cerebrovascular adverse events were discussed. Following a comprehensive risk-benefit assessment, dronabinol was initiated. The off label use of dronabinol was carried out in accordance with the Declaration of Helsinki and Good Clinical Practice guidelines.

The medical rationale behind this decision lay in the known positive anxiolytic, pain-relieving and calming effect of cannabinoids. As presented in **Figure 1**, during the following year pharmacological treatment could be reduced from six to three psychotropics including a low dose of dronabinol (6–8 drops dronabinol/day equivalent to 4.9–6.7 mg dronabinol). At the same time, the patient’s emotional state improved, while disruptive behavior, aggression, and sedation decreased significantly. So far, the patient is still in nursing care at her son’s home. She has never regained mobility and fluent speech. However, she shows little positive emotional response and allows nursing care without anxious or aggressive behavior. In the course of treatment with dronabinol, no side effects or adverse events occurred.

## Discussion and Conclusion

Summarizing, the reported case allows for the following conclusions: AD-associated NPs are common and burdensome for patients, their caregivers, and nursing care. Although there are no FDA-approved drugs for NPS (with the exception of short-term treatment with risperidone for severe aggression), it is common clinical practice to prescribe psychotropic medication such as antipsychotic drugs or sedatives to control symptoms in the long term. Some new-generation antipsychotics show modest efficacy in improving NPS, however, significant adverse effects and an increased mortality have been reported (8). Importantly, clinically significant and insufficiently treated NPS are associated with a more rapid disease progression, an increase of caregiver burden, and higher mortality.

Therefore, continuous nonpharmacological medical care as well as safe and effective alternative pharmacological treatments may have the potential to modify the course of the disease, lower costs, and improve quality of life of patients and their caregivers. In this case report, oral dronabinol turned out as promising candidate for treating behavioral symptoms such as agitation, aggression and anxiety even in a severe stage of AD (11–14). We are aware that our data have several limitations. The main limitation is that we report a single case and that our findings can therefore not be generalized. Further, our data allow no clear statement whether the introduction of dronabinol and/or the change of other psychopharmacological treatment improved the patient’s NPS and reduced the caregivers’ burden. However, a strength of this case report is the very long follow-up period of 11 years with a close and detailed recording of clinical data, medication history and NPs.

We assume that the observed positive effect of dronabinol on aggression and anxiety was mediated by the activation of the cannabinoid receptors 1 (CB_1_) and the activation of endocannabinoids. Prior studies reported that especially lower doses of THC may have anxiolytic effects (17, 18). Further, our findings corroborate those of Herrmann et al. who reported on a positive effect of the cannabinoid nabilone—a synthetic oral THC analog—on agitation in patients with AD (19). Our report underpins the need for randomized, controlled trials to explore the effect of cannabinoid receptor agonists on behavioral and psychological symptoms in patients in different stages of AD. Cannabinoids have a distinct pharmacologic profile that may offer an alternative pharmacologic approach to antipsychotics and sedatives for treating NPs in patients with AD. In addition, the beneficial effect on appetite and pain may significantly improve quality of life of AD-patients and their caregivers. Further research is needed to investigate the effects of different doses and types of cannabinoids in more detail. Especially in patients with severe AD, controlled clinical trials comparing cannabinoids with atypical antipsychotics are urgently needed.

## Data Availability Statement

All datasets generated for this study are included in the article.

## Ethics Statement

The study involving human participants was approved by the local ethical committee of Innsbruck Medical University, Austria. Written informed consent was obtained from the patient’s next of kin for the publication of any identifiable data.

## Author Contributions

MD reviewed and revised the manuscript and collected clinical data and obtained patient and caregiver consent. AH reviewed and revised the manuscript.

## Conflict of Interest

The authors declare that the research was conducted in the absence of any commercial or financial relationships that could be construed as a potential conflict of interest.
